# Circadian rhythms in pediatric craniopharyngioma

**DOI:** 10.3389/frsle.2023.1153144

**Published:** 2023-04-25

**Authors:** Dana Kamara, Stephanie J. Crowley, Valerie McLaughlin Crabtree, Donna Hancock, Yimei Li, Himani Darji, Joshua Semko, Merrill S. Wise, Thomas E. Merchant, Belinda N. Mandrell

**Affiliations:** ^1^Department of Psychology, St. Jude Children's Research Hospital, Memphis, TN, United States; ^2^Section of Pulmonary and Sleep Medicine, Department of Pediatrics, University of Colorado Anschutz Medical Campus, Aurora, CO, United States; ^3^Department of Psychiatry and Behavioral Sciences, Rush University Medical Center, Chicago, IL, United States; ^4^Division of Nursing Research, Department of Pediatric Medicine, St. Jude Children's Research Hospital, Memphis, TN, United States; ^5^Department of Biostatistics, St. Jude Children's Research Hospital, Memphis, TN, United States; ^6^Mid-South Pulmonary and Sleep Specialists, Memphis, TN, United States; ^7^Department of Radiation Oncology, St. Jude Children's Research Hospital, Memphis, TN, United States

**Keywords:** craniopharyngioma, pediatric brain tumor, sleep, circadian rhythm, melatonin, dim light melatonin onset (DLMO)

## Abstract

**Introduction:**

Craniopharyngioma is a brain tumor arising in the region of the hypothalamic-pituitary axis. Children and adolescents with craniopharyngioma have high survival rates, but often experience significant morbidity, including high rates of sleep disorders. Vulnerabilities to circadian disruption are present in this population, but little is known about circadian health.

**Methods:**

We present exploratory circadian findings from a prospective trial at a single center. Data presented here are from the baseline timepoint. Fifty-three patients between the ages of 7 and 20 years provided salivary melatonin samples, following surgical resection and prior to completion of proton therapy, when indicated. We estimated dim light melatonin onset (DLMO) and collected additional sleep data from actigraphy, overnight polysomnography, and the multiple sleep latency test.

**Results:**

Almost half of participants did not have a valid DLMO estimate during the sampling window, with most being above the threshold at the first sample timepoint. Those with greater disease severity variables (greater hypothalamic involvement and the presence of diabetes insipidus) were significantly more likely to have missed DLMO. For those with valid estimates, DLMO timing correlated with BMI and other sleep variables, including mean sleep latency values on the MSLT.

**Discussion:**

These findings suggest that a subset of those with pediatric craniopharyngioma may experience a phase advance and that this may relate to poorer prognostic indicators. Furthermore, circadian timing correlates with other sleep and health factors. Further research with earlier sampling is needed to better understand circadian rhythms in pediatric craniopharyngioma and associations with other health and disease variables.

## Introduction

Craniopharyngioma is a rare intracranial tumor located in the hypothalamic and pituitary region (e.g., Halac and Zimmerman, 2005; Müller, 2014). Approximately 6 to 10% of children with brain tumors have craniopharyngioma, with peak pediatric onset between 5 and 14 years of age. The typical treatment approach is a combination of surgical resection and radiation therapy (Müller et al., 2019). Survival rates for craniopharyngioma are very high, with a recent treatment study follow-up demonstrating an overall survival rate of 96% at 10 years (Edmonston et al., 2022). Despite high survival rates, quality of life may be substantially affected due to significant morbidity, including headaches, visual impairment, endocrine dysfunction, cognitive late effects, and sleep disturbances (e.g., Halac and Zimmerman, 2005; Müller, 2008; Khan et al., 2012; Fournier-Goodnight et al., 2017; Mandrell et al., 2020; Merchant et al., 2022).

Related to sleep disturbance, rates of narcolepsy and hypersomnia due to medical condition in pediatric craniopharyngioma are as high as 80%, despite the low prevalence of these conditions in the general population (e.g., Plazzi et al., 2018; Mandrell et al., 2020). Children and adolescents with craniopharyngioma who are overweight or obese are more likely to manifest hypersomnia or narcolepsy, with higher grade of hypothalamic tumor involvement associated with greater likelihood of a narcolepsy diagnosis (Mandrell et al., 2020). Children and adolescents with hypersomnia or narcolepsy are at risk for poor psychosocial functioning (Plazzi et al., 2018; Graef et al., 2020; Ingram et al., 2021), which further compounds poorer quality of life outcomes among youth with craniopharyngioma. High rates of sleep disorders are likely related to effects of the tumor location and treatment on nearby brain structures (i.e., optic chiasm, pituitary gland, hypothalamus) critical for sleep-wake regulation (España and Scammell, 2011).

Damage to the hypothalamic region also suggests likely disruptions to circadian rhythms, which are regulated by the suprachiasmatic nucleus (SCN) in the hypothalamus (Dibner et al., 2010). An examination of potential circadian rhythm disruption in patients diagnosed with craniopharyngioma is important to improve our understanding of sleep disturbance.

**Figure 1:**
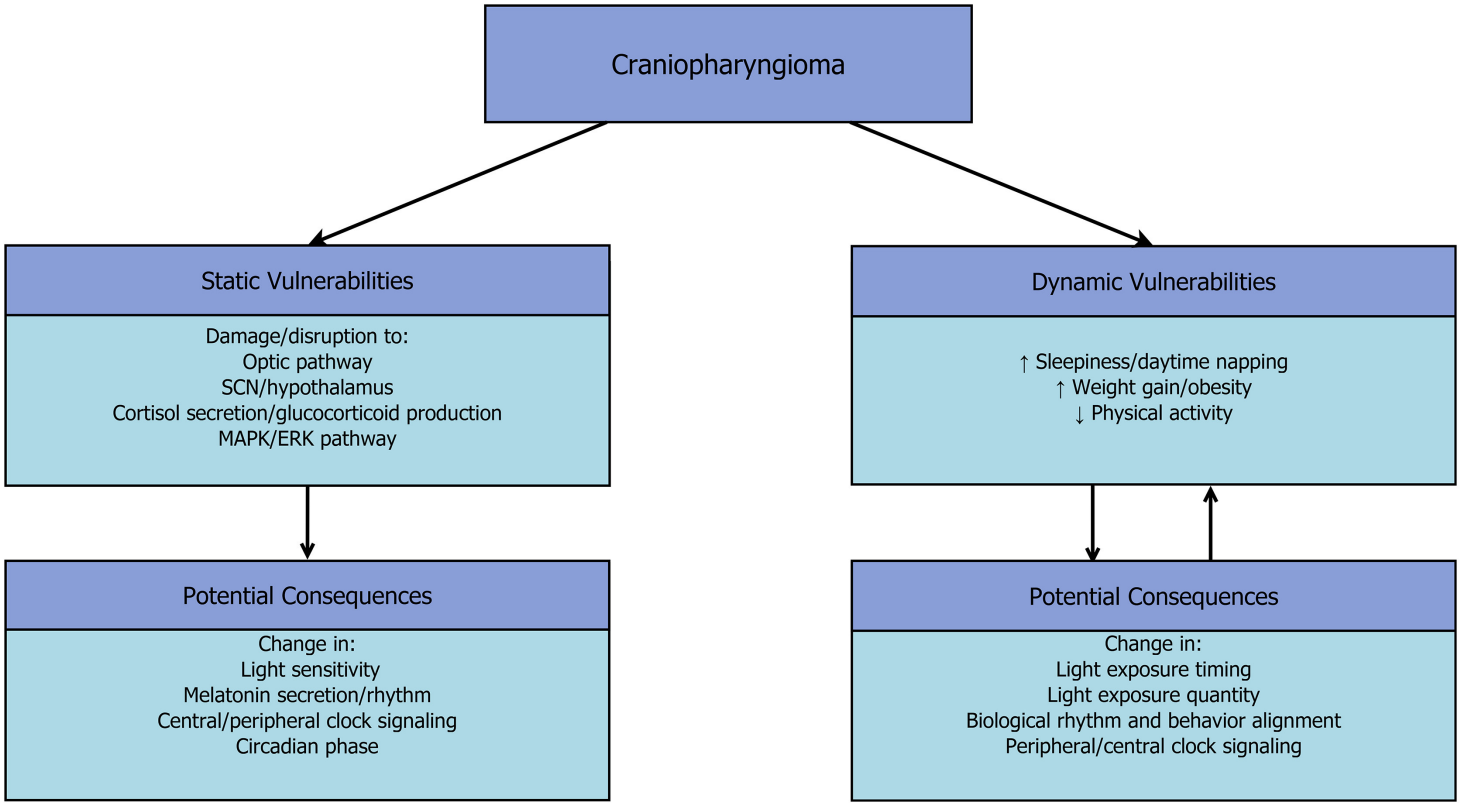
Theoretical mechanisms of circadian disruption in craniopharyngioma.

A theoretical overview of potential mechanisms of circadian rhythm disruption is outlined in Figure 1 and details both static and dynamic vulnerabilities to circadian disruption. Static vulnerabilities, or those that may be unchanging consequences of the tumor and/or surgical intervention, may include damage to the optic pathway, damage to the SCN, endocrine disruption, and changes to MAPK/ERK signaling pathways (Halac and Zimmerman, 2005; Müller et al., 2019; Hengartner et al., 2020). Damage to the optic pathway may cause changes in light sensitivity with downstream effects on circadian entrainment (e.g., Abbott et al., 2021), while damage to the SCN may directly affect timing of melatonin secretion (Dibner et al., 2010). Endocrine disruption, such as adrenal insufficiency, can alter the rhythm of cortisol secretion and other glucocorticoids with the potential for downstream effects on signaling and synchronization between the SCN and peripheral clocks (e.g., Chan and Debono, 2010). The potential for overactivity in the MAPK/ERK pathway is proposed in craniopharyngioma, and, if present, may lend vulnerability to circadian phase shifts (Kalkman, 2012; Hengartner et al., 2020).

Dynamic vulnerabilities may interact with and be further exacerbated by resulting circadian disruption. Behavioral changes, such as increased napping, decreased physical activity, and increased caloric intake/changes to meal timing may lead to disruption and misalignment between circadian timing and behavior. This may further contribute to weight gain and poor sleep health (e.g., Carskadon and Acebo, 2002; Culnan et al., 2021). Therefore, there is the theoretical potential for circadian disruption to arise through various mechanisms—although the effects of vulnerabilities on circadian function are yet to be demonstrated.

Thus far, a few studies have investigated the potential for circadian rhythm disruption in craniopharyngioma by measuring overall levels of melatonin, a hormone that helps establish circadian rhythmicity (Benloucif et al., 2008). Patients with craniopharyngioma have been found to have lower levels of melatonin than healthy controls, providing initial evidence that the rhythm of melatonin production may be disrupted (Lipton et al., 2009; Pickering et al., 2014). Furthermore, low melatonin levels are associated with higher self-reported sleepiness and additional sleep problems (as measured by actigraphy) such as decreased nighttime sleep duration and reduced sleep efficiency (Müller et al., 2002; Pickering et al., 2014).

While these findings are suggestive of potential circadian rhythm disruption, these studies have typically been small and/or included a wide age range from children to adults. Further study in a large sample of children and adolescents with craniopharyngioma would be beneficial for more precise characterization of circadian health in pediatric craniopharyngioma. In addition to measuring overall melatonin levels, the timing of melatonin secretion provides key information about circadian phase. Dim light melatonin onset (DLMO) reflects the timing of the rapid increase in melatonin secretion that occurs just prior to habitual bedtime (Crowley et al., 2006). If circadian phase is shifted (i.e., advanced or delayed), this could have implications for the understanding of sleep disturbance in craniopharyngioma and the development of effective treatments.

The present study is derived from an exploratory aim of a treatment protocol for pediatric craniopharyngioma. Details of the larger treatment study are provided below. The purpose of the exploratory aim was to examine circadian factors in pediatric craniopharyngioma. Within this aim, we had two study goals:

- To estimate DLMO values and phase angles of entrainment in our sample of children and adolescents with craniopharyngioma. Phase angles of entrainment demonstrate alignment between circadian phase and sleep timing and are defined as the time between DLMO and sleep onset and offset (Crowley et al., 2014).
- To examine correlations between DLMO timing and clinical variables, including hypothalamic involvement (HI), body mass index (BMI), Tanner stage, presence of a disorder of hypersomnolence, and additional sleep parameters, such as nighttime sleep duration, sleep onset latency, sleep efficiency, and wake after sleep onset.

Prior research on circadian factors and craniopharyngioma has been focused on melatonin levels, rather than timing of melatonin secretion. Therefore, our study hypotheses were largely exploratory. We hypothesized that DLMO timing would correlate with the presence of hypersomnia due to medical disorder or narcolepsy, sleep duration, and sleep efficiency, consistent with reported associations between low melatonin and other sleep parameters (self-reported daytime sleepiness, decreased nighttime sleep duration, reduced sleep efficiency; Müller et al., 2002; Pickering et al., 2014). We also predicted that DLMO timing would correlate with mid-sleep time, which reflects a behavioral estimate of circadian timing (Terman et al., 2001; Roenneberg et al., 2003). We hypothesized that DLMO timing would correlate with BMI based on prior reported findings of an association between melatonin levels and BMI in pediatric craniopharyngioma (Müller et al., 2002). Finally, we expected DLMO to be later with more mature Tanner stage, consistent with the typical circadian shift in adolescence (Carskadon, 2011; Crowley et al., 2018).

## Materials and methods

### Participants

Participants (*n* = 53), ages 7–20 years, were recruited from a single center institutional treatment protocol for pediatric craniopharyngioma, a Phase II trial of proton therapy for craniopharyngioma (ClinicalTrials.gov Identifier: NCT01419067; PI: TM, DO, Ph.D.). Inclusion criteria were as follows: (1) craniopharyngioma diagnosed by histology, cytology or neuroimaging; (2) Patient age of 21 or younger at time of diagnosis. For the salivary melatonin collection procedure, only patients 7 and older were asked to provide samples. Patients were excluded if: (1) they had a prior history of fractionated radiation therapy; (2) they had prior treatment with intracystic P-32, intracystic bleomycin or radiosurgery; or (3) if they were pregnant.

All eligible patients agreed to participate. They were enrolled following surgical resection and prior to proton therapy (when indicated). Measures were collected at the baseline timepoint, defined as within 12 weeks of initiation of therapy or observation. At baseline, treatments recommended by St. Jude physicians for co-occurring conditions, such as endocrine or sleep conditions, had not yet been initiated. Participants provided melatonin samples 24.8 ± 15.3 (average ± SD) days prior to starting proton therapy. Recruitment of patients took place between 2011 and 2017.

All procedures were approved by the Institutional Review Board of St. Jude Children's Research Hospital. Parents and adult participants provided consent, and pediatric participants provided assent for participation in the study. Fifty-nine participants met inclusion criteria and were asked to provide saliva samples for the study. Five participants did not provide sufficient samples for analysis (further information below), and one took a melatonin supplement when providing a sample; therefore, the final study sample included 53 participants.

### Procedures

#### Demographic information

Demographic and health characteristics were compiled as part of the larger treatment protocol. Hypothalamic involvement (HI), visual field deficit, and visual acuity were all graded categorically. HI was coded based on neuroimaging, with grade 0 (no HI), grade 1 (anterior HI), and grade 2 (anterior and posterior HI; Müller et al., 2011). Visual field deficit was characterized as grade 0 indicating no deficit, grade 1 indicating unilateral field deficit, and grade 2 indicating bilateral field deficit. Finally, visual acuity was graded as grade 0 (no deficit), grade 1 (unilateral reduced vision), or grade 2 (bilateral reduced vision). All participants had light perception. We also included information about Tanner stage, body mass index (BMI), and whether participants had diabetes insipidus (DI), which is a common endocrine complication of craniopharyngioma and/or surgical intervention (Edmonston et al., 2022).

#### Saliva collection

We followed the procedure for obtaining in-home saliva collection previously outlined in Mandrell et al. (2018). Prior to collection, a study nurse provided instructions for saliva collection and storage methods to participants and their caregivers. Families were instructed to collect saliva samples at hourly intervals, beginning 3 h prior to habitual bedtime and ending 1 h after habitual bedtime. Samples were collected by passive drool through a straw into collection tubes under dim light conditions (<30 lux, i.e., bedside light or television). Families were instructed to have the participant avoid consuming all food and beverages 30 min prior to collection, and to avoid certain foods (bananas, chocolate), caffeinated beverages, certain medications (e.g., melatonin, NSAIDS), and heavy meals on day of collection.

Saliva samples were color-coded according to their collection time and stored overnight in a labeled box in the family's freezer. The following morning, participants and their caregivers delivered the samples to the study nurse. The samples were stored in a freezer at −80°C until they were shipped to Salimetrics^®^ for analysis. Melatonin concentration levels were then extracted from saliva samples by Salimetrics^®^ with an ELISA assay. In order to have adequate quantity for the assay, the recommended quantity for each saliva test sample was 100 μl; quantities less than this were considered of insufficient quantity.

DLMO was calculated based on a 4 pg/mL threshold (Carskadon et al., 1997). Through linear interpolation among collection timepoints, we estimated the time at which melatonin concentration exceeded and remained above 4 pg/mL. We calculated phase angles of entrainment by measuring the time interval between DLMO and average sleep onset/waketime derived from actigraphy data (see below).

#### Actigraphy

To assess sleep-wake patterns, patients were instructed to wear a Micromini Sleep Watch^®^ on their wrist for 5–7 nights (Ambulatory Monitoring Inc., Ardsley, NY). Data were recorded in 1-min epochs and scored using the Sadeh algorithm (Sadeh et al., 1994). Actigraphy data for each participant was included if there were at least two nights of actigraphy data available before or after the night of saliva collection. The night of saliva collection was excluded as collection procedures delayed sleep onset. We averaged actigraphy data for each participant (range of two to five nights) to obtain the following sleep variables: average sleep onset, waketime, midsleep time, total sleep time, sleep efficiency, sleep onset latency, and wake after sleep onset. We also calculated the standard deviations of these variables to provide a measure of variability in sleep patterns. For variability analyses, participants with at least three nights of actigraphy data were included. Actigraphy data were manually inspected by investigators for the presence of non-24 or other circadian rhythm disorders. None were found based on manual inspection.

#### PSG/MSLT

Participants completed an overnight polysomnography (PSG) and multiple sleep latency test (MSLT) the following day. These studies were typically performed beginning within 30 min of the subject's usual bedtime. We followed a standard MSLT protocol with 4 or 5 nap opportunities at 2-h intervals (Littner et al., 2005; Aurora et al., 2012). Variables derived from the MSLT results include the presence of hypersomnia due to medical disorder or narcolepsy due to medical disorder as diagnosed by a board-certified sleep physician, mean sleep latency for naps (MSLTsol), and number of sleep onset REM periods during naps (SOREMP). All sleep disorder diagnoses were made using diagnostic criteria from the *International Classification of Sleep Disorders, 3rd edition* (ICSD-3; (American Academy of Sleep Medicine, 2014) applied by a board-certified sleep medicine physician who examined the patient during a sleep clinic evaluation and interpreted the PSG and MSLT using accepted criteria.

### Data analysis

#### Primary analyses

We calculated DLMO timing for our sample as described above and were able to estimate DLMO for approximately half of our sample (*n* = 28). We also calculated phase angles of entrainment to sleep onset and wake time.

We examined Spearman's rank correlations between DLMO values and the following variables: age, BMI, Tanner stage, HI, sleep onset latency (SOL) during the MSLT. We also examined Spearman's rank correlations between DLMO values and the averages and standard deviations of the following actigraphy variables: wake after sleep onset (WASO), sleep efficiency (SE), SOL, sleep duration, and sleep onset time, midsleep time, and wake time. Finally, we examined Wilcoxon Mann-Whitney U tests to compare whether DLMO differed between those with or without disorders of hypersomnolence, with or without narcolepsy, with or without diabetes insipidus, and dichotomized puberty status (Tanner stage 1 vs. stages 2–5).

#### Exploratory analyses

We were interested in understanding whether there were demographic or clinical differences between subgroups for whom we were and were not able to estimate DLMO. Therefore, patient characteristics were summarized by descriptive and clinical characteristics for both the DLMO and missed DLMO subgroups. Associations between measurement of DLMO and other variables were examined with the following analyses: Fisher's exact test—sleep disorder category (hypersomnia, narcolepsy, neither) and race (Black, White, Other); chi-square test—sex, presence of diabetes insipidus, and dichotomized puberty status (i.e., pre-pubertal, pubertal), Cochran Armitage trend test (HI, visual acuity, visual field deficit, Tanner stage). Due to non-normal distribution of the following continuous variables, and a small sample size, Wilcoxon Mann-Whitney U tests were performed to detect the median difference in age, BMI, MSLTsol and SOREMP between the DLMO and Missed DLMO subgroups.

In addition to demographic and clinical characteristics, we examined differences in sleep behavior and variability in sleep timing between the subgroups by comparing the means and standard deviations of the actigraphy variables with Wilcoxon Mann-Whitney U tests. All statistical analyses were completed with SPSS version 25 (IBM Corp., Armonk, NY) and (SAS Institute, Cary, NC).

## Results

### Estimation of DLMO

For the participants for whom we could not estimate DLMO (*n* = 25), 19 had melatonin levels already above the 4 pg/ml threshold at time of first measurement, 2 had levels that remained below the threshold for all measurements, and 4 had levels that rose above the threshold, but did not remain above the threshold.

### Demographic, sleep, and health characteristics

Participant demographic, sleep, and health characteristics are presented in Table 1. The age of participants ranged from 7 to 20 years (*M* = 11.70 ± 3.74). Most participants (83%) had a disorder of hypersomnolence. Overnight polysomnography data are presented in Table 2.

**Table 1:** Demographic, sleep, and health characteristics.

**Variable**	**Full sample**	**DLMO**	**Missed DLMO**	***p***
*N*	53	28	25	
**Age**				0.957
Mean (SD)	11.70 (3.74)	11.79 (3.97)	11.60 (3.56)	
Median (IQR)	11.00 (9.00–15.00)	11.00 (8.50–15.00)	10.00 (9.00–15.00)	
**Sex**, ***n*** **(%)**				0.487
Female	26 (49.1%)	15 (53.6%)	11 (44.0%)	
Male	27 (50.9%)	13 (46.4%)	14 (56.0 %)	
**Race**, ***n*** **(%)**				0.810
Asian	2 (3.8%)	2 (7.1%)	0 (0%)	
Black or African American	11 (20.8%)	6 (21.4%)	5 (20.0%)	
Multiracial	5 (9.4%)	2 (7.1%)	3 (12.0%)	
Other	1 (1.9%)	1 (3.6%)	0 (0%)	
White	34 (64.2%)	17 (60.7%)	17 (68.0%)	
**Sleep disorder**, ***n*** **(%)**				0.838
Hypersomnia	25 (47.2%)	15 (53.6%)	10 (40.0%)	
Narcolepsy	19 (35.9%)	9 (32.1%)	10 (40.0%)	
No Diagnosis	7 (13.2%)	3 (10.7%)	4 (16.0%)	
Missing	2 (3.8%)	1 (3.6%)	1 (4.0%)	
**Hypothalamic involvement**, ***n*** **(%)**				0.021^*^
0	7 (13.2%)	7 (25.0%)	0 (0%)	
1	16 (30.2%)	8 (28.6%)	8 (32.0%)	
2	30 (56.6%)	13 (46.4%)	17 (68.0%)	
**Visual field deficit**, ***n*** **(%)**				0.876
0	33 (62.3%)	19 (67.9%)	14 (56.0%)	
1	4 (7.5%)	1 (3.6%)	3 (12.0%)	
2	14 (26.4%)	8 (28.6%)	6 (24.0%)	
Missing	2 (3.8%)	0 (0%)	2 (8.0%)	
**Visual acuity**, ***n*** **(%)**				0.341
0	37 (69.8%)	22 (78.6%)	15 (60.0%)	
1	5 (9.4%)	1 (3.6%)	4 (16.0%)	
2	4 (7.5%)	2 (7.1%)	2 (8.0%)	
3	6 (11.3%)	3 (10.7%)	3 (12.0%)	
4	1 (1.9%)	0 (0%)	1 (4.0%)	
**Tanner stage**, ***n*** **(%)**				1.000
1 (pre-pubertal)	32 (60.4%)	17 (60.7%)	15 (60.0%)	
2–5 (pubertal)	18 (34.0%)	9 (32.1%)	9 (36.0%)	
Missing	3 (5.7%)	2 (7.1%)	1 (4.0%)	
**BMI**				0.304
Mean (SD)	23.09 (5.06)	22.04 (4.18)	24.27 (5.76)	
Median (IQR)	22.00 (19.40–26.50)	22.00 (19.40–24.80)	22.80 (19.35–29.35)	
**DI**				0.040^*^
Present, *n* (%)	26 (49.1%)	10 (35.7%)	16 (64.0%)	
**MSLTsol**				0.288
Mean (SD)	9.56 (9.76)	9.33 (5.46)	9.82 (13.03)	
Median (IQR)	7.60 (3.69–13.00)	9.25 (4.70–13.10)	6.00 (3.50-11.00)	
**SOREMP**				0.300
Mean (SD)	1.16 (1.36)	1.00 (1.36)	1.35 (1.37)	
Median (IQR)	1.00 (0–2.00)	0 (0–2.00)	1.00 (0–3.00)	

Sample is separated into those with valid dim light melatonin onset (DLMO) estimates and those without. P-values indicate comparisons in demographic variables between DLMO and Missed-DLMO subgroups. Due to low frequencies in some racial categories, differences in race were examined as Black, White, Other, with Other including Asian, multiracial, and other. BMI, body mass index; DI, diabetes insipidus; MSLTsol, mean sleep latency measured on multiple sleep latency test; SOREMP, sleep onset rapid eye movement periods measured on multiple sleep latency test.

* *p* < 0.05.

**Table 2:** Polysomnography findings.

**Variable**	**Full sample**
*N*	51
**Total sleep time (mins)**	
Mean (SD)	473.02 (57.63)
**Sleep efficiency (%)**	
Mean (SD)	89.85 (6.87)
**Sleep onset latency (mins)**	
Mean (SD)	23.23 (41.72)
**Apnea-hypopnea index**	
Mean (SD)	1.29 (1.87)
**Periodic limb movements**	
Mean (SD)	8.39 (11.66)

#### Subgroup differences

There were two significant health characteristic differences identified between the subgroups of DLMO and missed DLMO. A higher grade of hypothalamic involvement was associated with higher likelihood of missed DLMO, Cochran Armitage trend test, *p* = 0.021. The presence of diabetes insipidus was associated with higher likelihood of missed DLMO (χ^2^ test, *p* = 0.040).

In terms of sleep behavior, the missed DLMO subgroup had a longer median SOL (averaged over all actigraphy days) of 32.10 min than the DLMO subgroup median SOL of 17.75 min (*U* = 730, *p* = 0.005). The missed DLMO subgroup also had a more variable SOL of 19.38 min compared to 6.53 min in the DLMO subgroup, when the standard deviation of SOL was examined (*U* = 262, *p* = 0.043). Additional comparisons between missed DLMO and DLMO subgroups for sleep behavior are available in Table 3.

**Table 3:** Actigraphy comparisons between DLMO and missed DLMO.

**Variable**	**DLMO**	**Missed DLMO**	***P***
	Median (IQR)	Median (IQR)	
**Start time**			
Average	21:49:30 (21:29:40–22:06:30)	21:44:35 (21:07:30–22:03:30)	0.559
SD	00:31:17 (00:19:08–00:48:52)	00:37:42 (00:22:53–01:19:56)	0.508
**End time**			
Average	7:11:15 (6:48:30–7:27:30)	7:21:05 (6:40:18–7:47:06)	0.439
SD	00:30:59 (00:28:29–00:41:08)	00:47:14 (00:30:12–01:08:20)	0.058
**Mid time**			
Average	2:26:27 (2:03:45–2:26:27)	2:27:39 (2:06:35–2:57:35)	0.801
SD	00:26:08 (00:22:02–00:35:04)	00:39:34 (00:25:52–00:50:52)	0.061
**Duration (mins)**			
Average	560.10 (528.50–591.67)	575.90 (528.27–609.20)	0.593
SD	47.30 (31.32–64.59)	64.79 (46.97–99.38)	0.083
**Efficiency (%)**			
Average	91.19 (83.60–95.49)	87.36 (83.55–91.14)	0.154
SD	4.31 (3.25–7.39)	5.33 (3.25–8.10)	0.767
**SOL (mins)**			
Average	17.75 (10.33–29.50)	32.10 (20.80–41.17)	0.005^*^
SD	6.53 (4.11–19.42)	19.38 (12.50–26.34)	0.043^*^
**WASO (mins)**			
Average	46.30 (26.00–88.50)	66.83 (49.23–89.50)	0.069
SD	23.27 (19.91–52.64)	30.19 (19.04–40.83)	0.922

Sample is separated into those with valid dim light melatonin onset (DLMO) estimates and those without. Sample sizes for analyses with averages are *n* = 50, while sample sizes for analyses with standard deviations are *n* = 39 due to requirement for at least 3 days of consecutive actigraphy data for variability analyses. Differences between both the average values and standard deviations (SDs) of sleep variables for DLMO and Missed DLMO were examined using the Mann-Whitney U-test. Analysis for standard deviation of end time conducted with Brunner-Munzel test due to unequal variances between the groups. Mins, minutes; SOL, sleep onset latency; WASO, wake after sleep onset. P-values indicate comparisons in sleep variables between DLMO and Missed-DLMO subgroups.

* *p* < 0.05.

### DLMO timing and associations with sleep and health characteristics

DLMO and phase angles of entrainment are presented in Table 4. DLMO timing and BMI were positively correlated (*r*_*s*_ = 0.48, *p* = 0.012), as were DLMO timing and sleep onset latency on the MSLT (*r*_*s*_ = −0.55, *p* = 0.003). DLMO timing was positively correlated with average waketime on actigraphy (*r*_*s*_ = 0.40, *p* = 0.042), and average midsleep time (*r*_*s*_ = 0.47, *p* = 0.015). DLMO timing was negatively correlated with standard deviation of sleep onset time (*r*_*s*_ = −0.47, *p* = 0.049). There were no other significant associations between DLMO timing and examined sleep or health variables. There were no significant differences in DLMO timing based on sleep disorder status, puberty status, or the presence of diabetes insipidus.

**Table 4:** DLMO and phase angles of entrainment.

**Variable**	***n***	**Mean (*SD*)**
DLMO (hours)	28	21.11 (1.23)
DLMO to bedtime phase angle (minutes)	26	40.88 (72.53)
DLMO to wake time phase angle (minutes)	26	604.38 (76.99)

DLMO, dim light melatonin onset. Ns for DLMO to bedtime and DLMO to wake time phase angles are 26 because two participants with valid DLMO estimates did not have valid actigraphy data.

## Discussion

In this study, we examined circadian rhythms among children and adolescents with craniopharyngioma by estimating DLMO. DLMO was missed for almost half our sample (*n* = 25), with most patients in the missed DLMO group above the threshold at the time of first collection. For these patients, it is possible that they had an earlier circadian phase. There is some evidence to suggest that at least a small subset of individuals with craniopharyngioma (2 of 14 patients in one study) show a phase advanced rhythm (Pickering et al., 2014). Circadian phase advances are also consistent with our clinical observations that patients often describe an early morning preference. Anecdotally, even adolescents with craniopharyngioma commonly report a morning preference, which is atypical for this age group.

The likelihood of missed DLMO was not associated with any examined demographic variables. However, it was associated with greater disease severity, as defined by hypothalamic involvement and presence of diabetes insipidus. Diabetes insipidus, unless present prior to surgery, reflects extensive surgical resection (Edmonston et al., 2022). This suggests that capturing DLMO within the sampling window was less likely for those with more severe encroachment on brain structures from the tumor and subsequent surgical intervention(s) (Halac and Zimmerman, 2005; Müller et al., 2019).

We also examined whether missed DLMO may have been related to sleep health variables. Surprisingly, missed DLMO did not relate to the presence of a central disorder of hypersomnolence. This was an unexpected finding, although it could have been due to the very high rates (83%) of these conditions in our sample, limiting variability. We found that patients in the missed DLMO subgroup took longer to fall asleep at night and had a more variable sleep onset latency, as measured by actigraphy, than those in the DLMO subgroup. It is not clear why patients in the missed DLMO subgroup had longer and more variable SOL. Many of these patients showed melatonin values that were above threshold at the beginning of sampling, which suggests that their DLMO may have occurred earlier (phase advanced). However, we do not know for certain if this is the case without more data to confirm a phase advance. It is also possible that those in the missed DLMO subgroup have circadian dysregulation overall, which could be directly or indirectly (e.g., napping behavior) contributing to lengthened sleep onset latency. Unfortunately, we did not gather data on napping behavior.

For those with a valid DLMO estimate, our hypotheses were partially supported. Although DLMO timing was not associated with the presence or absence of hypersomnia or narcolepsy, later DLMO timing was associated with increased sleepiness severity on the MSLT (reflected by a shorter average sleep onset latency). That we observed significant differences when data were looked at continuously, but not categorically, is not entirely surprising given the very high rates of disorders of hypersomnolence in our sample. In contrast to our hypotheses, we did not find associations between DLMO timing and sleep duration or efficiency. However, later DLMO timing was associated, as one might expect, with later midsleep and wake times. Later DLMO timing was also associated with decreased variability in actigraphy sleep onset time. Consistent with our hypothesis that DLMO timing and BMI would be associated, we found that patients with higher BMIs had later DLMO timing. Finally, we did not find an association between DLMO timing and age or Tanner stage, which is inconsistent with well-established findings of circadian shift in adolescence (Carskadon, 2011; Crowley et al., 2018). This may reflect disrupted sleep/wake regulation in this sample of children and adolescents with craniopharyngioma; however, a lack of association between DLMO timing and Tanner stage may simply reflect a power issue as the majority of participants with valid DLMO estimates were in Tanner stage 1.

Overall, our findings suggest possible circadian phase advance in a subset of children and adolescents with pediatric craniopharyngioma and that circadian timing is related to additional health factors. It will be important to further study circadian rhythms in these patients in order to inform clinical practice. In clinical settings, DLMO is not routinely used, but actigraphy may be used as a proxy to identify patients with advanced phase or other forms of circadian disruption. Note, however, that validation of using actigraphy to estimate circadian phase has not been systematically tested in this patient group. If indicated, targeted interventions, such as bright light therapy and melatonin administration, may be beneficial (Abbott et al., 2020). Circadian health is important for broader sleep and overall health, and consideration of circadian health may improve health and daytime function for patients.

### Strengths/limitations

This project had a few limitations. Our inability to capture DLMO for almost half our sample limits our ability to draw conclusions about circadian timing in this population. In addition, actigraphy collection was too brief to identify circadian rhythm abnormalities. We also did not recruit control participants. Although there are healthy norms published for DLMO and phase angles of entrainment among children and adolescents (Crowley et al., 2006), there were too many methodological differences to facilitate adequate comparison between our samples. In addition, due to the nature of our center, patients may have arrived from another time zone prior to completing the assessments. We did not collect data on whether families had traveled from another time zone or whether they had sufficient time to adjust. We cannot disentangle tumor effects from surgical effects as enrolled participants had already completed surgical resection, when indicated. We also did not have information available about circadian rhythm or chronotype prior to surgery. Finally, the nature of this study is cross-sectional which limits our ability to understand circadian changes over time.

This project also had several strengths. Given the rarity of pediatric craniopharyngioma, we were able to recruit a relatively large cohort of patients. Our sample was well-characterized with all patients recruited prior to proton therapy. We gathered data with robust and well-validated sleep-wake and circadian rhythm measures (salivary melatonin, actigraphy, PSG/MSLT). Finally, we successfully collected saliva samples outside of the lab, which should have permitted a more normative sleep/wake schedule.

### Future directions

Given challenges with DLMO estimation, it will be important for future studies to begin sampling earlier to capture DLMO in these patients. Even more ideal would be to sample melatonin comprehensively for at least 24 h to determine the daily pattern of secretion. To assist with feasibility of 24-h collection, urine sampling may be used instead of saliva samples (Benloucif et al., 2008). However, precise information about phase changes are more difficult to estimate with urine sampling. Core body temperature is another circadian measure that may be useful; findings in small samples of adults with craniopharyngioma show disruption to core body temperature rhythm (Joustra et al., 2014; Foschi et al., 2017). We will explore this measure in an upcoming project. While the understanding of circadian health in pediatric craniopharyngioma is in preliminary stages, it will be important to compare children and adolescents with pediatric craniopharyngioma to healthy controls to determine whether disruption is present and to better understand individual differences related to circadian outcomes.

Future studies should also evaluate how potential circadian disruption interacts with other sleep health characteristics, including daytime sleepiness and sleep variability. In this study, we found some significant associations between sleep variability and valid estimation of DLMO, as well as DLMO timing. We measured sleep variability over 3–5 days, and this may have limited our capacity to fully examine these associations. Variability in sleep timing is a key sleep health construct and relates to other health outcomes (Meltzer et al., 2021). Future studies should include a more comprehensive assessment of variability by examining sleep patterns for at least 1–2 weeks. Circadian and sleep health are not stagnant constructs. It will be important to include longitudinal assessment to better understand how circadian and broader sleep health outcomes change over time, and better understand how circadian health interacts with other health domains (e.g., physical, emotional, neurocognitive). In a future study, we will evaluate longitudinal effects on DLMO in this patient population.

## Conclusions

Our study findings suggest that a subset of children and adolescents with craniopharyngioma may have phase advance and that this relates to poorer prognostic indicators (e.g., hypothalamic involvement of the tumor). For those with valid DLMO estimates, timing correlated with sleepiness and BMI. Further study of circadian health in pediatric craniopharyngioma is needed to refine treatments and improve care for patients.

## Data availability statement

The raw data supporting the conclusions of this article will be made available by the authors, without undue reservation.

## Ethics statement

The studies involving human participants were reviewed and approved by St. Jude IRB. Written informed consent to participate in this study was provided by the participants' legal guardian/next of kin.

## Author contributions

TM is the principal investigator for the prospective treatment protocol from which circadian data was collected. TM, MW, BM, and DH completed sleep and health assessments and collected all data for the project. Specific project conceptualization was completed by BM, VC, DK, and SC. SC provided circadian expertise and consultation. Data management and analysis was completed by DK, YL, HD, and JS. All authors contributed to manuscript development and editing. All authors contributed to the article and approved the submitted version.
